# FRAILTY AND THE COMPARATIVE EFFECTIVENESS AND SAFETY OF SGLT2I AND GLP1-RA IN OLDER ADULTS WITH TYPE 2 DIABETES

**DOI:** 10.1093/geroni/igz038.2158

**Published:** 2019-11-08

**Authors:** Elisabetta Patorno, Ajinkya Pawar, Seoyoung Kim, Dae Kim

**Affiliations:** 1 Brigham and Women’s Hospital and Harvard Medical School, Boston, Massachusetts, United States; 2 Brigham and women’s hospital, Boston, Massachusetts, United States; 3 hebrew SeniorLife, Boston, Massachusetts, United States

## Abstract

We conducted a 1:1 propensity score-matched retrospective cohort study of 87,218 patients with type 2 diabetes (mean age, 71.5 years [standard deviation, 5.1]) initiating a SGLT2i or a GLP1-RA in Medicare data. We estimated HRs (95% CIs) for a composite cardiovascular endpoint and severe hypoglycemia comparing the two treatments in the entire population and by the CFI-based frailty subgroups. Compared with GLP1-RA, SGLT2i were associated with similar rates of the composite cardiovascular endpoint (HR, 0.94 [95% CI, 0.86-1.03]) and severe hypoglycemia (0.87 [0.71-1.07]) over a mean follow-up of 8.6 months. The rate of composite cardiovascular endpoint was not meaningfully different between SGLT2i and GLP1-RA across non-frail (1.33 [0.80-2.23]), pre-frail (0.96 [0.85-1.08]), and frail patients (0.87 [0.73-1.04]). Similarly, the rate of severe hypoglycemia was not meaningfully different between the two treatments among non-frail (0.97 [0.20-4.80]), pre-frail (0.83 [0.64-1.08]), and frail patients (0.95 [0.67-1.34]).

